# A Review of the Role of Extracellular Polymeric Substances (EPS) in Wastewater Treatment Systems

**DOI:** 10.3390/ijerph191912191

**Published:** 2022-09-26

**Authors:** Lei Huang, Yinie Jin, Danheng Zhou, Linxin Liu, Shikun Huang, Yaqi Zhao, Yucheng Chen

**Affiliations:** 1 Key Laboratory of the Three Gorges Reservoir Region’s Eco-Environment (Ministry of Education), College of Resources and Environment, Southwest University, Chongqing 400715, China; 2 Chongqing Key Laboratory of Agricultural Resources and Environment, Chongqing 400716, China

**Keywords:** extracellular polymeric substances, wastewater treatment, microbial aggregation, biological characteristics, contaminant removal, sludge properties

## Abstract

A review of the characterization and functions of extracellular polymeric substances (EPS) of microbial aggregates in biological wastewater treatment systems is presented in this paper. EPS represent the complex high-molecular-weight mixture of polymers excreted by microorganisms generated from cell lysis as well as adsorbed inorganic and organic matter from wastewater. EPS exhibit a three-dimensional, gel-like, highly hydrated matrix that facilitates microbial attachment, embedding, and immobilization. EPS play multiple roles in containments removal, and the main components of EPS crucially influence the properties of microbial aggregates, such as adsorption ability, stability, and formation capacity. Moreover, EPS are important to sludge bioflocculation, settleability, and dewatering properties and could be used as carbon and energy sources in wastewater treatment. However, due to the complex structure of EPS, related knowledge is incomplete, and further research is necessary to understand fully the precise roles in biological treatment processes.

## 1. Introduction

Activated sludge is the main key in the common biological process for industrial and municipal wastewater treatment plants [1]. Most of the microorganisms are present in the form of microbial floccules, such as sludge flocs, biofilms, and granules. The major components of microbial aggregates are the microorganisms and the extracellular materials, known as extracellular polymeric substances (EPS). EPS are complex extracellular polymers with high molecular weight, which are metabolic products of microorganisms and result from effluent organic matter and microbial lysis or hydrolysis [2]. Furthermore, EPS usually serve as a protective layer against the harsh external surrounding [3] and a major composition of microbial floccules for keeping the sludge cells together [4]. 

Polysaccharides and proteins are the two main components of EPS [5,6,7]. Other components, such as humic acids, nucleic acids, lipids, and uronic acids, have also been detected in certain amounts in EPS [5,7,8]. In addition, there were some inorganic components in EPS from various matrixes [8]. The contents and compositions of EPS in sludge flocs are related to many factors, including extraction methods, wastewater type, sludge origins, and operational conditions. EPS contents may be closely related to the growth phase of the bacteria and increase with incubation time increasing in the exponential growth phase. However, EPS contents also could decrease with incubation time increasing in the stationary phase [9].

EPS were proved to have the ability to absorb and biodegrade certain substances. The main components in EPS were found to influence the properties of microbial floccules, such as transfer, surface hydrophilicity/hydrophobicity, and aggregate stability [7]. Recently, the spatial distribution of EPS has been demonstrated to affect the bioflocculation, settling, and dewatering properties of activated sludge [10]. Because EPS play a role in mass transfer between cells and environments, they can also influence microbial metabolisms and pollutant removal [7]. EPS have a large number of functional groups, such as carboxyl, phosphate, amine, sulfhydryl, pheaffectnol, and hydroxyl. Most of the functional groups are negatively charged at neutral pH and capable of forming organometallic complexes with metal ions. Therefore, EPS can be used as biosorbents for the removal and recovery of heavy metals in certain industrial wastewater [11]. Xiong et al. indicated that EPS were widely used in the food, cosmetics, and pharmaceutical industries because of their good physical, rheological, and safety properties [12]. EPS with a high content of fucose could be regarded as valuable chemicals for industrial applications, particularly in cosmetics and pharmaceuticals areas [13]. Therefore, investigating the roles of EPS in wastewater treatment systems, especially in the activated sludge process, has attracted significant interest. Although several review articles have summed up the roles of EPS, the previous research advanced needs to be summarized and analyzed [7]. 

The aim of this review is to provide an overview of the characteristics and functions of EPS in biological wastewater treatment systems. The contents are as follows: (1) to clarify the structure, distribution, and extraction methods of EPS; (2) to identify the biological characteristics of EPS, such as stability, adhesion ability, and biodegradability; (3) to describe the roles of EPS on contaminants removal such as organic matter, nitrogen, phosphorus, and metal ions; (4) to discuss the effects of EPS on sludge flocculation, settleability, and dewatering capacity.

## 2. Fundamentals of EPS

### 2.1. EPS Structure and Distribution

EPS have a three-dimensional structure with a gel-like and highly hydrated matrix; the microorganisms are embedded and more or less immobilized in EPS [14]. Owing to the distinct structure, EPS possess a vast surface area and carry numerous functional groups (e.g., carboxyl, phosphoric, amine, and hydroxyl) [15], significantly affecting the physico-chemical characteristics of sludge flocs, such as adhesion, hydrophobicity, settling, and dewatering. Two forms of EPS can be subdivided outside of microorganisms cells, including bound EPS (sheaths, capsular polymers, condensed gels, loosely bound polymers, and attached organic materials) and soluble EPS (soluble macromolecules, colloids, and slimes) [16]. Although the interaction between soluble EPS and cells is weak, the soluble EPS are found to have impacts on microbial activity and sludge properties [17]. However, the research on soluble EPS is limited, and the EPS mentioned in this review without being specified are bound EPS. Bound EPS surrounding bacteria or in sludge flocs likely possess a dynamic double-layered structure where tightly bound EPS (TB-EPS) forms an inner layer, and loosely bound EPS (LB-EPS) diffuses in the outer layer [18]. LB-EPS are highly hydrated and tend to form a dispersible and loose slime layer without a significant edge (dispersible part). Sludge settleability and dewaterability were found to be more strongly correlated with LB-EPS than TB-EPS [19], while TB-EPS were found to have effective flocculation activity compared to LB-EPS [20].

### 2.2. Methods of Extracting EPS

Several EPS extraction methods can be employed from various wastewater sludge (biofilm and aerobic or anaerobic activated sludge). Centrifugation is the most common extraction method for soluble EPS. Notably, lots of extraction methods for bound EPS have been generated, and new approaches are being developed [21]. These extraction methods can be classified as physical methods and chemical methods, listed in Table 1. The physical extraction methods usually adopt external forces, which are created by ultrasonic, centrifugation, or heating, to encourage the EPS to detach from cells and then dissolve in solution. The chemical extraction methods involve adding chemical compounds to disrupt the binding interactions between the EPS and the cells to accelerate the dissolution of the EPS. In general, the extraction efficiencies of the physical extraction methods are lower than those of the chemical extraction methods. As LB-EPS loosely bind with cells, mild methods, such as high-speed centrifugation, heating at low temperatures, or high-rate shear, should be utilized to prevent TB-EPS release. In addition to various physical methods, chemical methods, and enzyme treatments, the extraction of TB-EPS usually adopts more harsh combination methods, including CER + sonication, alkaline + heating, and formaldehyde + heating.

## 3. Biological Characteristics of EPS

### 3.1. Stability

In biological treatment systems, the stability of microbial floc is important to solid/liquid separation [35]. EPS exist in the structure of microbial floccules and interact with cells by polymer entanglement, electrostatic interaction, and ion bridging, as well as hydrogen bonds and van deer Waal’s force. These intermolecular interactions contribute to the stability of microbial aggregates. This means that higher EPS contents lead to higher stability in sludge [36]. Sludge has been reported to have a multiple-layer structure with two independent parts. The outer layer is dispersible and could be extracted readily, and the internal part is stable, where the non-readily extractable EPS glue the residual sludge cells. Thus, closer relationships with sludge stability exist among the readily extractable EPS [37].

### 3.2. Adhesion Ability

The formation of biofilm in a liquid environment is related to micro-surface characteristics. EPS present on cell surfaces could enhance microbial deposition [38]. Some functional groups in EPS contribute to microbial adhesion onto surfaces [39,40]. In the previous study, the number of adherent bacteria on the sludge surface was found to be reduced after EPS removal [41], and EPS-rich strains have stronger adhesion to bacteria than the EPS-deficient strains under similar surface charge conditions [42]. Thus, the adhesion of cells created by EPS can help to generate bioflocculation [43].

### 3.3. Biodegradability

Carbon and energy from EPS can be used by activated sludge [44]. Proteins and polysaccharides are the main substances in EPS, and the degradation enzymes for these polymers are adequate in biological wastewater treatment reactors. The excreted EPS were found to be utilized by bacteria for metabolic activity when there was a nutrient shortage [45,46]. However, some studies showed that some EPS could not be degraded by microbes [16]. EPS located in the inner layer of aerobic granular sludge were reported to be biodegradable, but the situation was on the contrary in the outer layer [47]. Although some EPS can be used as carbon resources, the degradation can cause deflocculation in the activated sludge system. Moreover, the non-degradable EPS can be discharged along with the effluent and bring negative effects on the effluent quality [48].

## 4. Contaminant Removal

### 4.1. Organic Matter Removal

Synthetic organic matter is extensively used in many fields, and the unintended release into the environment poses a potential risk to human health and ecological systems [49]. EPS, as a kind of synthetic organic matter, play an important role in organic pollutant removal [50]. EPS contain large quantities of negatively charged functional groups with strong capabilities to adsorb organic pollutants [51], including phenanthrene [52], benzene [53], humic acids [54], dye [55], and antibiotics [56]. A previous study reported that more than 60% of benzene, toluene, and m-xylene could be absorbed by EPS and that only a small part was performed by cells [57]. Because the binding strength and capability of proteins are higher than that of humic substances, soluble EPS with more proteins have a greater ability to bind organic pollutants than the bound EPS [58]. 

It was pointed out that EPS was important for organic pollutant adsorption, especially for some special organic matter, such as antibiotics [55]. Large amounts of antibiotics from hospitals and pharmaceutical factories have been discharged into the sewage plant every year [59,60]. Sorption was regarded as the primary way to remove antibiotics in activated sludge systems [61,62], and the adsorption capacity would decrease significantly after EPS removal [63]. Notably, sulfamethazine, one typical antibiotic, could be effectively removed by EPS adsorption, which was beneficial for the subsequent biodegradation [64].

### 4.2. Nitrogen and Phosphorous Removal

Nitrogen and phosphorous are common nutrients in domestic wastewater [65]. The biological phosphorus and nitrogen removal process has been developed for treating wastewater and protecting water against eutrophication [66]. The activated sludge process is the wildly used and well-established biological nutrient removal process [67]. Notably, the EPS of sludge also play an important role in phosphorus and nitrogen removal because of the special roles in mass transfer [68,69,70]. The differences in surface functional groups of EPS lead to variations in hydrophilicity and hydrophobicity, influencing nutrient removal [71].

Cloete and Oosthuizen indicated that phosphorous could be accumulated significantly in sludge EPS, and the role of EPS in phosphorous removal could not be negligible in biological wastewater treatment systems [70,72]. The removal process of phosphorous by EPS through transformation and transportation was considered to occur simultaneously with the phosphorus-accumulating organism metabolism and phosphorous precipitation [70,71,73]. Wang et al. indicated that 27% of phosphorus were adsorbed by EPS in the denitrifying phosphorus removal process [74].

EPS adsorption also has positive effects on biological nitrification/denitrification [74], especially for promoting the adsorption of ammonium [75]. Respectively, 32.94% and 72.29% of total nitrogen were removed by EPS adsorption in the processes of heterotrophic denitrification and anammox [69]. However, as EPS are highly complex, the roles of nutrient removal need to be further studied. In particular, knowledge of pollutant removal pathways by EPS in activated sludge systems is very limited [71,73].

### 4.3. Metal Ion Removal

Ecotoxicological risks of heavy metals are widely investigated and have been a global concern [76]. The existence of heavy metals in the aquatic system can be detrimental to various living species because these materials are non-biodegradable and tend to accumulate in living organisms, causing diseases and disorders [77]. Owing to the non-biodegradable characteristics of heavy metals, biological sludge in municipal or industrial wastewater facilities can be effectively used in heavy metal removal processes [78]. Sludge consists of numerous organisms and several organic substances, such as EPS and cell flocs [11], and has been proven to adsorb heavy metal ions in wastewater [79,80,81,82]. EPS also display acid-base and metal-ion binding properties [83,84,85,86]. The investigation has shown the adsorption-desorption behaviors of Hg(II) and Sb(V) on EPS [78]. Other authors also studied the sorption of Pb(II) and Cd(II) by EPS extracted from pure bacterial strains or activated sludge and showed EPS adsorbed over 80% Pb(II) and 30% Cd(II) at pH 7 [5]. Mayer et al. found that Ca(II) and Mg(II) could be removed in activated sludge [87]. Furthermore, a previous study reported that some trace elements (e.g., Cu, Zn, and Ni) might be trapped by the organic matrix of EPS [88].

Functional groups of EPS, such as carboxyl, phosphoric, amine, sulfhydryl, phenolic, and hydroxyl groups [89], harbored by cell walls, govern the metal-ion sorption [85,86,90]. These functional groups represent potential binding sites for the sequestration of metal ions [91]. Proteins, carbohydrates, and nucleic acids in EPS all have the ability to bind with heavy metals [92]. Proteins and humic substances in EPS were both strong ligands for Cu(II), and further investigation showed that Cu(II) was bound with oxygen atoms in the carboxyl groups of EPS [80]. Additionally, the abundant charged groups in EPS can react with metal ions [69].

Several parameters, such as temperature, ionic strength, pH, biosorbent size, biosorbent dosage, initial solute concentration, and agitation rate [93], will influence the ability of biosorbents to bind with metal ions. Because the metal affinity order to EPS changes with pH [94], the biosorption for metal ions can be significantly influenced by pH [95]. For example, the number of EPS binding sites for Cu(II), Pb(II), and Cd(II) could increase with pH increasing. It was reported that EPS produced by *Parapedobacter* sp. ISTM3 strain removed 70% Cr(VI) at pH 4.0 and 95% Cr(VI) at pH 5.0 [96]. However, knowledge of how pH affects EPS binding sites is very limited [94]. Higher temperatures usually enhance sorption due to the increased surface activity and kinetic energy of the solute [97]. EPS have different binding capacities for different metal ions. The binding ability to Pb(II) is better than that to Cd(II) [5], while Cu(II) can be adsorbed more easily than Zn(II) [98]. 

The mechanisms of metal sorption, such as chelation, ion exchange, proton exchange, coordination, and precipitation, are involved during metal and EPS interactions [5,99]. The adsorption of heavy metals onto the unfractionated and hydrophobic EPS could be better described by the Langmuir isotherm, while Freundlich models are more suitable for hydrophilic EPS [98]. However, further work is needed to obtain a full understanding of the precise roles of EPS on heavy metals removal in biological treatment systems.

## 5. Effect of EPS on Sludge Properties

The activated sludge process is widely used, and the production of waste sludge is an inevitable drawback inherent in the system. Serious pollution problems will be caused if large quantities of waste sludge are not appropriately treated and disposed of [100]. During sludge treatment, sludge settling and dewaterability are the main obstacles faced by wastewater treatment plants [101]. EPS are the important components of activated sludge and play a specific role in sludge bioflocculation, settling, and dewatering properties [11].

### 5.1. Effects of EPS on Flocculation Ability

The flocculation ability of microbial aggregates is essential to achieve high quality and low turbidity in effluent. The effects of shear rate on the floc size distribution and the correlation between flocculation dynamic parameters (such as collision efficiency and breakage rate coefficient) and velocity gradient have been investigated widely in previous studies. In fact, the zeta potential of flocs and the organic matter contents of EPS are also important for activated sludge flocculation [11,48,102,103,104]. Bioflocculants, such as microbial EPS, are natural organic macromolecule substances, flocculating suspended bacterial cells, solids, and colloidal solids [105]. The highly hydrated gel matrix produced from EPS keeps the cells together [106], while the interactions between EPS and cells have significant effects on microbial flocculation ability, showing EPS of flocs as the key factor [107]. Adding a certain dose of LB-EPS or TB-EPS to the sludge solution after EPS extraction could induce the aggregation of sludge, and the aggregation capacity of sludge was restored to more than 80% [102,103,104].

The flocculation ability of microbial aggregates decreases with the total EPS increasing and increases with protein contents increasing or humic substance contents decreasing [108]. Proteins constitute more than 43% of the total amount of EPS, while humic substances constitute 15–42% [108]. However, no obvious relationships were found between nucleic acid and sludge characteristics. Proteins and polysaccharides in EPS significantly affected the flocculability of sludge, and the sludge had good flocculability when the proteins and polysaccharides in EPS were less than 21.55 and 12.27 mg/g VSS, respectively [109]. Thus, the proportion of the main EPS components might have effects on microbial flocculation [110]. Xing et al. also emphasized that EPS easily extracted were more beneficial for flocculation. Some studies reported that the concentration of EPS was an important influencing factor for bioflocculation [111], and too high or too low a concentration of EPS would lead to a reduction in flocculation activity [20]. Recently, studies have demonstrated that the spatial distribution of EPS also affected the bioflocculation, settling, and dewatering properties of activated sludge [10].

EPS promote or bate cell aggregation with flocculation and settling. The flocculation dynamic parameters are linearly correlated with the EPS content and zeta potential. The floc size will gradually increase, while the flocculation balance factor will decrease with increasing EPS content or decreasing absolute values of the zeta potential. Although high EPS favored large flocs aggregation, the flocs containing high EPS were more prone to large-scale fragmentation [112]. Although EPS was essential to biofloc formation, excessive EPS in the form of LB-EPS would weaken cell attachment and deteriorate the activated sludge floc structure, thereby resulting in poor biosolid–water separation [113].

Liu et al. indicated that both LB-EPS and TB-EPS substantively affected sludge aggregation, but the contributions to sludge aggregation were different [48]. LB-EPS fraction did not play an important role in bioflocculation, but the active part (i.e., TB-EPS fraction), considered to be the most active fraction in wastewater-activated sludge, was responsible for the high flocculation rate [114]. Similarly, Bezawada et al. found that TB-EPS demonstrated the highest flocculation activity and dewaterability compared to LB-EPS [20], due to the presence of a large number of macromolecules (330–1200 kDa) and trivalent cations [114].

### 5.2. Effects of EPS on Settling Ability

Sludge settling properties are often regarded as the bottleneck in terms of upgrading or increasing the capacity of wastewater treatment plants [115]. It was reported that the number of EPS was more important to the sludge settleability than the composition [114]. Some studies reported that sludge settled faster with lower EPS [116], whereas others reported the opposite [117]. Poxon and Darby [118] revealed that EPS contained a high proportion of bound water, producing highly porous sludge flocs with lower density, which led to poor sludge settleability. In addition, the increasing EPS concentration enhanced the cell surface charge and then increased the repulsive forces among cells, generating a decrease in activated sludge settleability [48]. 

The various components of EPS have different effects on the settling ability of activated sludge. The production of highly porous flocs with low density due to the increase of LB-EPS could induce irreducible water into the aggregates [113], expressing the negative effects on sludge settleability. The SVI of microbial aggregates will increase with EPS contents increasing. However, the effects of the major components of EPS on the settleability of microbial aggregates are not clear. Proteins in EPS were found to have a positive relationship with SVI [119], but carbohydrates had no significant effects [120]. Additionally, no significant correlation had been observed between SVI and nucleic acid [109]. 

### 5.3. Effects of EPS on Dewatering Ability

Dewatering is a crucial process in sludge treatment and involves significant technical challenges. Reducing the water content of sludge cake is proven to be an efficient method of decreasing transportation and disposal costs. However, the highly hydrated and negatively charged EPS in sludge colloids can bind a large volume of water, i.e., bound water, within the sludge flocs and affect the charge and stability of the flocs. EPS are usually regarded as the key factor in the thickening and dewatering process of sludge [121,122]. There are two binding mechanisms of EPS and water molecules, including electrostatic interactions and hydrogen. The former played a major role in the permanent dipole of the water for the functional groups of EPS, and the latter rose the main function in hydroxyl groups of EPS and water molecules [123]. All these prevent sewage sludge from dewatering.

In general, the reason why the increase in EPS results in poor sludge dewatering ability may be that the steric resistance generated by EPS prevents contractions among cells. The macromolecules in EPS cause a large amount of water retained in the sludge floc and increase the amount of interstitial water. EPS can also form a stable gel preventing water seepage from the pores of flocs, which deteriorates the dewatering ability of the sludge [44]. Ni et al. reported that aerobic digestion increased dissolved EPS and reduced the sludge dewatering ability [124]. The effects of EPS on sludge dewatering ability may be related to the EPS contents and components. A previous study has shown that proteins with high water-holding capacity may induce an increase in dissolved EPS [125]. Therefore, the purpose of improving sludge dewatering ability can be achieved by reducing the protein portion in sludge EPS [126]. In addition, increasing the proportion of carbohydrates in EPS also would enhance the sludge dewatering [126]. Zhang et al. found that the proteins and polysaccharides in EPS may control dewaterability, and the sludge had good dewatering ability when the proteins and polysaccharides in EPS were no more than 21.55 and 12.27 mg/g VSS, respectively [109]. However, some studies have shown that with the increase of EPS content, the sludge dewatering ability improves [125]. Thus, the interaction of EPS and the dewatering ability of sludge needs to be further studied in the future, and the specific role remains elusive so far. 

Short-chain fatty acids (SCFAs), generated from waste activated sludge (WAS) [127], have been considered a good carbon source needed for biological nutrient removal [128]. Enhancing SCFA production from WAS fermentation can substantially reduce the cost of biological nutrient removal and effectively reduce WAS volume. Recently, the relation of EPS with sludge reduction has been examined. Zhao et al. indicated that EPS in sludge were the main matrix to produce SCFAs and helped to achieve sludge reduction [129]. However, the specific mechanism and process are still unknown and limited research is available.

## 6. Findings

EPS are complex extracellular polymers with high molecular weight, including bound EPS (subdivided into LB-EPS and TB-EPS) and soluble EPS. EPS have various biological properties such as stability, adhesion capacity, and biodegradability. Given the different structural traits of LB-EPS and TB-EPS, different extraction methods need to be adopted. As the LB-EPS loosely bind with cells, mild methods, such as high-speed centrifugation, heating at low temperatures, or high-rate shear, should be utilized to prevent TB-EPS release. TB-EPS are usually extracted by harsh methods, including CER + sonication, alkaline + heating, and formaldehyde + heating.

EPS have been found to play an important role in activated sludge treatment systems for pollutants removal such as organic matter, nitrogen, phosphorus, and metal ions. In terms of organic removal, the presence of EPS will help to remove phenanthrene, benzene, humic acids, dyes, and even antibiotics by adsorption, beneficial for biodegradation in subsequent treatments. The adsorption of EPS also presents positive influences on biological nitrogen and phosphorus removal. The organic matrix in EPS can sequester some heavy metal ions, and the functional groups of EPS, including carboxyl, phosphate, amine, sulfhydryl, phenol, and hydroxyl groups, can also provide potential binding sites for target metal ions. 

EPS can generate significant effects on the bioflocculation, settling, and dewatering properties of sludge. Although both LB-EPS and TB-EPS contribute substantially to sludge aggregation, different functions have been found. LB-EPS have adverse effects on sludge settlement, while the presence of TB-EPS facilitates sludge to exhibit the highest flocculation activity. The effects of EPS on sludge dewatering ability may be related to the EPS contents and components. Reducing the protein portion or increasing the proportion of carbohydrates in EPS will enhance the sludge dewatering. Additionally, EPS in sludge are the main matrix to produce SCFAs and help to achieve sludge reduction.

## 7. Conclusions

The activated sludge process is the common biological process used in municipal and industrial wastewater treatment plants. As the complex high-molecular-weight mixture of polymers produced by microorganisms and the protective barrier for cells against the harsh external environment, EPS are the important components of activated sludge. The major components of EPS are polysaccharides, proteins, humic acids, and nucleic acids. Several EPS extraction methods have been sorted out and analyzed, including physical and chemical methods, and different extraction methods can be adopted according to different research purposes. EPS have strong capabilities to adsorb organic pollutants, nutrients, and heavy metal ions in wastewater treatment systems, because of the large quantities of negatively charged functional groups involved in the matrix. The complex components in EPS are found to crucially affect the properties of microbial aggregates, such as adsorption ability and stability. Moreover, EPS are important to sludge bioflocculation, and settling and dewatering properties. EPS can also be used by sludge floc as sources of carbon and energy in wastewater treatment. 

## 8. Recommendations

As EPS are highly complex, knowledge regarding the generation mechanism of EPS is far from complete, and further work is needed to obtain a full understanding of the precise roles of EPS in biological treatment systems. In particular, research about how EPS influence the mass transmission between cells and the external environment is extremely limited. Furthermore, the effect of various components of EPS on its functionality remains unknown and limited research is available. At present, many studies are being conducted on EPS, but further information on EPS is crucial to ensure its success as a commercial product for real-world application. 

## Figures and Tables

**Table 1:** Different TB-EPS extraction methods.

Methods		Mechanism	Features	Disadvantages	References
Physical	Centrifugation	EPS are separated from cell surface and then dissolve to solution under the centrifugal force.	Comparatively less cell lysis. Separate the soluble EPS from the cellular biomass.	The lowest amounts of EPS are extracted. Bound EPS cannot be extracted significantly.	[8,11,22,23,24]
	Heating	EPS dissolution will be accelerated by enhancing molecular movement with heating.	Loosen the sludge structure by heating. Separate EPS from microbial cells easily.	Significant lysis and cells disruption. Partial EPS hydrolysis.	[11,22,25,26]
	Sonication	EPS of biofilm matrix are extracted under different impulsive pressures created by sonication.	Effectively disintegrate sludge flocs and release enzymes. Mild and effective. EPS are separated using the shear force generating from ultrasound and the pressure formed by the cavity rupture.	EPS stripping is not complete and the number of extractions is low. Disruption of the extracellular matrix.	[11,22,27,28,29]
	Sonication/centrifugation	EPS will dissolve into solution under the impulsive pressure created by the sonication and centrifugal force.	Mild and effective. Widely used method. Ultrasound and centrifugation techniques are repeatedly used to extract different grades of EPS.	Disruption of the extracellular matrix and partial cell breakage.	[22,30]
Chemical	Acidic treatment	EPS are fallen away from the cell surface, as the interaction between EPS and cells is disrupted by the repulsive force.	Rich in chemical groups (hydroxyl, peptide bond, polysaccharides, phosphate, and sulfur functional groups).	The extraction efficiency is low.	[8,11,22]
	Alkaline treatment	Alkaline treatment with NaOH addition causes the groups to be ionized, resulting in a strong repulsion between EPS and cells.	Effectively sever cell lysis and the disruption of macromolecules.	Neither extract more EPS nor get more chemical groups.	[8,11,22]
	CER	CER removes the divalent cations resulting in EPS falling apart.	High extraction efficiency. Widely used method. The products obtained from the processing facilitate subsequent analysis.	Insignificant cell lysis using low amount of CER.	[8,11,22,31]
	EDTA	EPS matrix will fall apart, because divalent cations for the cross-linking of charged compounds are removed using EDTA.	Cause a low degree of cell lysis. Mild and acceptable extraction efficiency.	Form complexes with EPS generating interfere in the colorimetric analysis.	[8,11,22,32]
	Enzymatic extraction	The carbohydrate and protein-hydrolyzing enzymes are used to disrupt the structure of sludge and dissolve EPS.	Stable humus contents in the extracted EPS. Mild and effective.	Specific for polymers. Represent only a minority of EPS. Underestimation of the total polymer.	[11,22,33]
	HCHO/NaOH	HCHO reduces the cell lysis caused by NaOH addition.	Extract carbohydrate, protein and uronic acid for all sludges. The highest amounts of EPS extracted from sludges.	Formaldehyde alters the structure and properties of proteins in EPS. Change the polymer composition at pH > 9.	[11,29,34]

## Data Availability

Not applicable.
